# Direct observation and manipulation of hot electrons at room temperature

**DOI:** 10.1093/nsr/nwaa295

**Published:** 2020-12-15

**Authors:** Hailu Wang, Fang Wang, Hui Xia, Peng Wang, Tianxin Li, Juzhu Li, Zhen Wang, Jiamin Sun, Peisong Wu, Jiafu Ye, Qiandong Zhuang, Zaixing Yang, Lan Fu, Weida Hu, Xiaoshuang Chen, Wei Lu

**Affiliations:** State Key Laboratory of Infrared Physics, Shanghai Institute of Technical Physics, Chinese Academy of Sciences, Shanghai 200083, China; University of Chinese Academy of Sciences, Beijing 100049, China; State Key Laboratory of Infrared Physics, Shanghai Institute of Technical Physics, Chinese Academy of Sciences, Shanghai 200083, China; University of Chinese Academy of Sciences, Beijing 100049, China; State Key Laboratory of Infrared Physics, Shanghai Institute of Technical Physics, Chinese Academy of Sciences, Shanghai 200083, China; University of Chinese Academy of Sciences, Beijing 100049, China; State Key Laboratory of Infrared Physics, Shanghai Institute of Technical Physics, Chinese Academy of Sciences, Shanghai 200083, China; University of Chinese Academy of Sciences, Beijing 100049, China; Henan Key Laboratory of Diamond Optoelectronic Materials and Devices, School of Physics and Engineering, Zhengzhou University, Zhengzhou 450001, China; State Key Laboratory of Infrared Physics, Shanghai Institute of Technical Physics, Chinese Academy of Sciences, Shanghai 200083, China; University of Chinese Academy of Sciences, Beijing 100049, China; State Key Laboratory of Infrared Physics, Shanghai Institute of Technical Physics, Chinese Academy of Sciences, Shanghai 200083, China; State Key Laboratory of Infrared Physics, Shanghai Institute of Technical Physics, Chinese Academy of Sciences, Shanghai 200083, China; University of Chinese Academy of Sciences, Beijing 100049, China; Henan Key Laboratory of Diamond Optoelectronic Materials and Devices, School of Physics and Engineering, Zhengzhou University, Zhengzhou 450001, China; School of Microelectronics, Shandong University, Jinan 250100, China; State Key Laboratory of Infrared Physics, Shanghai Institute of Technical Physics, Chinese Academy of Sciences, Shanghai 200083, China; University of Chinese Academy of Sciences, Beijing 100049, China; State Key Laboratory of Infrared Physics, Shanghai Institute of Technical Physics, Chinese Academy of Sciences, Shanghai 200083, China; University of Chinese Academy of Sciences, Beijing 100049, China; Department of Physics, Lancaster University, Lancaster LA1 4YB, UK; School of Microelectronics, Shandong University, Jinan 250100, China; Department of Electronic Materials Engineering, Research School of Physics and Engineering, The Australian National University, Canberra, ACT 2601, Australia; State Key Laboratory of Infrared Physics, Shanghai Institute of Technical Physics, Chinese Academy of Sciences, Shanghai 200083, China; University of Chinese Academy of Sciences, Beijing 100049, China; State Key Laboratory of Infrared Physics, Shanghai Institute of Technical Physics, Chinese Academy of Sciences, Shanghai 200083, China; University of Chinese Academy of Sciences, Beijing 100049, China; State Key Laboratory of Infrared Physics, Shanghai Institute of Technical Physics, Chinese Academy of Sciences, Shanghai 200083, China; University of Chinese Academy of Sciences, Beijing 100049, China

**Keywords:** hot electrons, valley transfer, photogating, scanning photocurrent mapping

## Abstract

In modern electronics and optoelectronics, hot electron behaviors are highly concerned, as they determine the performance limit of a device or system, like the associated thermal or power constraint of chips and the Shockley-Queisser limit for solar cell efficiency. To date, however, the manipulation of hot electrons has been mostly based on conceptual interpretations rather than a direct observation. The problem arises from a fundamental fact that energy-differential electrons are mixed up in real-space, making it hard to distinguish them from each other by standard measurements. Here we demonstrate a distinct approach to artificially (spatially) separate hot electrons from cold ones in semiconductor nanowire transistors, which thus offers a unique opportunity to observe and modulate electron occupied state, energy, mobility and even path. Such a process is accomplished through the scanning-photocurrent-microscopy measurements by activating the intervalley-scattering events and 1D charge-neutrality rule. Findings here may provide a new degree of freedom in manipulating non-equilibrium electrons for both electronic and optoelectronic applications.

## Teaser

A distinct approach to artificially (spatially) separate hot electrons from cold ones, which thus provides a new degree of freedom in manipulating nonequilibrium electrons for both electronic and optoelectronic applications.

## INTRODUCTION

In all existing transistors, electrons are easily warmed up to a temperature higher than the crystal lattice, and are denoted as non-equilibrium or hot electrons [1]. When flowing through the conduction channel, these hot electrons collide with the host lattice and transfer energies to the latter inevitably. It makes a transistor continuously give off heat to the environment and accounts for the heat-dissipation constraints of chips [2,3]. Similarly, hot carriers in solar cells were commonly considered to cause great loss (∼42%) of efficiency [4–6], because these hot carriers, typically generated by absorbing high-energy photons (above the bandgap of the semiconductor), tend to dissipate excess energies through the relaxation process [7,8]. Resolving those problems, nowadays, should rely on a direct observation and thorough understanding of hot carrier dynamic and kinetic processes, which, however, could be a great challenge [9]. Fundamentally, difficulties in experimental approaches to such a microscopic process arise from the fact that hot electrons are only separated from cold ones in the momentum-energy space but behave the same way (similar transmission path or acceleration process) as cold electrons in real space at most times [10]. It means the output electrical signal can only reflect an average conductance of those energy-differential electrons [11].

Efforts lately have been devoted to imaging plasmon-induced hot electrons with various approaches, like photoconductive atomic force microscopy [12], pump-probe spectroscopy [13,14] and surface chemistry modification [15,16] for tracing the hot electron-related photocurrent or surface molecules reduction/generation. Yet, these approaches cannot be easily applied to other systems (like transistors and other state-of-the-art electronic devices) due to the very specific application scenarios: surface plasmon and hot electron transfer at heterointerface [12–17]. The lack of experimental information also accounts for the problems in physical modeling and analyzing. For example, in quite a long period of time, an average velocity of electrons is the only information that can provide for the theoretical analysis of hot electrons, which dramatically increases the difficulties in resolving such typical non-equilibrium problems [10,11,18].

Herein, we develop a distinct and simple approach to characterizing hot electron behaviors in both momentum and real spaces. The study was performed based on a semiconductor nanowire (NW) transistor, since it confines charge carriers for a quasi-one-dimensional (1D) transport (rather than a zigzag motion in bulk counterpart), that will significantly enhance the chances of locating hot electrons, for example by their unique mobility, velocity or transport pathway. Note that semiconductor NW is also one of the most promising conduction-channel materials in the post-Moore era [19,20]; the 3D design allows gate electrodes to closely control the current flow, for example, a higher on-off ratio and lower power consumption when compared to the rival planar-MOSFET and FinFET [19]. Another key method of distinguishing hot electrons is scanning-photocurrent-microscopy

measurements (SPCM), a non-disruptive photoelectric characterization method that is commonly employed to confirm junction location [21–23], electrical-contacts configuration [24], the origin of photoresponse [25,26], etc. Here, SPCM is used to activate intervalley scattering events as well as the 1D charge neutrality rule, as a means of spatial separation of hot electrons from cold ones.

## RESULTS AND DISCUSSION

**Figure 1.**
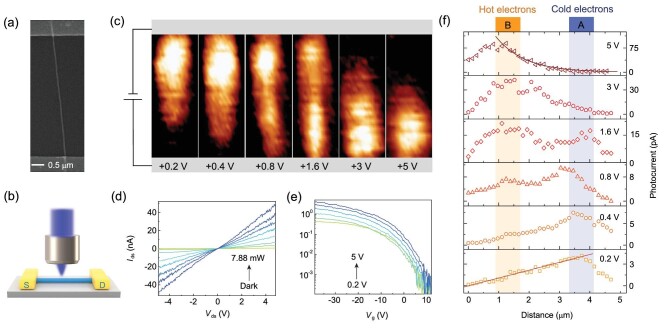
Electrical and photoelectric properties of a GaAs NW transistor. (a) SEM image of a back-gated GaAs NW (p-doped) transistor, ∼100 nm in diameter and ∼4.7 μm in channel length. (b) Schematic picture of the SPCM measurement setup. (c) SPCM images of a GaAs NW transistor at increasing bias-voltages on the bottom electrodes as indicated. The top electrode is kept ground. The photocurrent magnitude is represented by the color scale that increases from black (0) through orange to white (1). (d) Linear *I*_ds_ − *V*_ds_ curves in the dark and under 450 nm laser illumination. (e) Semi-logarithm *I*_ds_ − *V*_g_ characteristics at different *V*_ds_. (f) Photocurrent profiles along the axial direction of the NW extracted from the SPCM images. The solid brown (red) line is exponentially (linearly) fitted to the photocurrent versus distance at *V*_ds_ = 5 V and 0.2 V.

Figure 1a shows the scanning electron microscopy (SEM) image of a back-gated p-type gallium arsenide nanowire (GaAs NW) transistor, ∼100 nm in diameter and ∼4.7 μm in channel length. During the device fabrication process, NWs have been physically transferred onto a Si/SiO_2_ (280 nm) substrate, followed by a chemical etching process (to remove the native oxide layer on the GaAs NW surface). Right after that, source and drain electrodes (15 nm Cr/45 nm Au) were prepared by electron-beam lithography, thermal evaporation, lift-off and metallization processes; and the back gate was contacted by Au on the heavily doped Si substrate (details in the Methods section). As depicted in Fig. 1d, the linear *I*_ds_ − *V*_ds_ curves in both dark and illuminated conditions indicate the excellent ohmic contacts between GaAs NW and the source/drain electrode. Meanwhile, transfer characteristic plots (Fig. 1e) confirm the dominant role of holes in NW conductance. All electrical and photoelectric characterizations in this work were performed at room temperature and ambient conditions.

As schematically shown in Fig. 1b, SPCM images were obtained by a focused laser spot (with 450 nm in light wavelength, ∼1 μm in spot size) scanning over the nanowire device (∼5 μm × 2 μm) with the output photocurrent recorded in real time. The movements of the laser spot in X and Y directions were controlled by two vibrating mirrors, as displayed in Supplementary Fig. S12. In general, SPCM experiments provide an optical probe to test the photoresponse of each segmented part of the nanowire transistor (more experimental details about SPCM measurement setup can be found in Supplementary Section 8). By this means, the dependence of photoresponse on the spatial localization is derived. Experimental results are shown in Fig. 1c. One will find that there are typical ‘hot spots’ in the SPCM images that shift from near the cathode (through the entire NW channel) to the anode with an increasing bias-voltage (here, to show the direction of carriers movement, we employ the ‘cathode’/‘anode’ to represent the holes/electrons collector, corresponding to the top/bottom electrode in Fig. 1c). Electric fields in the opposite direction have also been applied, where SPCM patterns are correspondingly reversed (see Supplementary Fig. S1). It helps to exclude the influence of non-uniform doping or asymmetric electrodes. SPCM measurements under very small voltages (10 and 20 mV, Supplementary Fig. S4) have also been performed to confirm the absence of space charge region and contact-related photocurrent effects, which have been observed in 2D materials [22,23,26]. Figure 1f shows the 1D photocurrent profiles taken from SPCM images, which give a quantitative description of such characteristics. At small bias-voltage (0.2 V), only one peak of photocurrent is located near the cathode (*x *≈ 3.8 μm, denoted as feature A). With the increase of biased voltage, however, another hot spot can be identified in the other direction (*x *≈ 1.2 μm, denoted as feature B), and starts to dominate the photocurrent response. Also, it should be noted that completely different decay-curves are observed in peak A and B. More specifically, the photocurrent degrades linearly from the peak position to both sides at *V*_ds _= 0.2 V (see the solid red fitting curve in the bottom panel of Fig. 1f), but it would decrease exponentially at *V*_ds_ = 5 V (see the solid brown fitting line in the top panel of Fig. 1f). This detailed information is critical to understanding the underlying physics, which will be discussed later.

**Figure 2.**
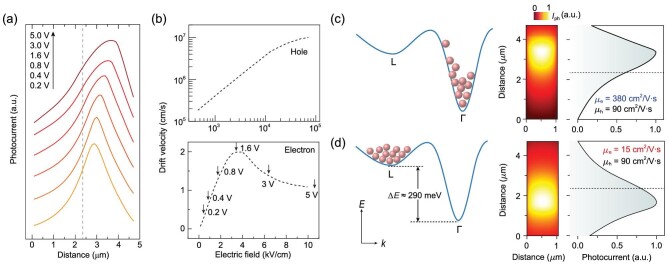
Physics and reproduction of the distinct hot spots in SPCM images. (a) Photocurrent profiles along the axial direction of NW by TCAD simulation. The mobility of electrons and holes in the simulation were 380 cm^2^/V · s and 90 cm^2^/V · s (from refs [29] and [30]). (b) Dependence of drift velocity on the electric field for the hole (reproduced with permission [32], copyright 1971, AIP Publishing) and electron (reproduced with permission [10], copyright 1980, Elsevier, Ltd.) in GaAs. The black arrows are added to show the electric field strength that applies in Fig. 1c and f. (c) and (d) First column: population of electrons in minimum Γ- and the upper L- valleys at a (c) weak and (d) high electric field. Second column: photocurrent mapping images obtained by the simulation at *V*_ds _= 1.6 V under the conditions of *μ*_e _= 380 cm^2^/V · s and *μ*_e_ = 15 cm^2^/V · s. Third column: simulated photocurrent profiles along the NW extracted from SPCM images. The midpoint of the NW is marked by black dashed lines.

The above phenomena deviate from previous observation, in which only one hot spot exists in SPCM images and it should not pass the midpoint of the channel (no matter how high or low the voltage bias is) [27,28]. A visual perspective of such a process is that there is only one optimal solution for photocarriers with opposite polarity to reach the anode and cathode simultaneously. It is determined by the charge neutrality principle, that can be written as ${\mu _e}Et + {\mu _h}Et\ = \ L$, where $E$ is the external electric field, $t$ is the carrier transit time, $L$ is the channel length, and ${\mu _e}$ and ${\mu _h}$ represent electron and hole mobility, respectively. Generally, the mobility of electrons is higher than that of holes, meaning electrons take on more path length (${P_e}$, equal to ${\mu _e}Et$). It explains why the hot spot should localize at a half-section of the NW [28]. A more quantitative description of this behavior relies on a rigorous simulation. Here we utilize Sentaurus TCAD, a commercial software package, to simulate the charge carrier transport in the NW transistor. Some key parameters [29,30] are summarized in Supplementary Table S1, and more details can be found in the Methods section. Figure 2a shows the calculated photocurrent profiles at different bias-voltages. Obviously, there is only one photocurrent peak (hot spot), and it is always close to one certain electrode (cathode here, electrode setup is identical to that of experiments). Note that a shift of photocurrent peak from near the midpoint (dashed line in Fig. 2a) to the cathode can be identified. It arises from a widened gap between electron and hole velocities/path length, $({{\mu _e} - {\mu_h}})E$, with an increased external electric field.

For transistors with reduced dimension, there is one critical issue that could not be neglected: the high field induces a hot electron effect, which may lead to negative differential mobility and drift velocity [31] in GaAs materials. Figure 2b shows the dependence of carrier drift velocity on the external electric field in bulk GaAs (from refs [10] and [32]); the black arrows are added in this work to show the electric field strength that applies in Fig. 1c and f (given the channel length of 4.7 μm). One will find that ∼3.5 kV/cm (1.6 V) is a turning point, after which the electron drift velocity/mobility degrades significantly. Such a character arises from the hot electron valley transfer, from Γ to L satellite valley (Fig. 2c and d, accompanied by a 13.5-fold increase in density-of-states effective mass) [33]. By contrast, the hole drift velocity would not saturate until the electric field reaches 10^4^ kV/cm, approximately four orders of magnitude higher than that of electrons. It means that the hole mobility is a constant within the scope of this study. In consideration of this effect, two kinds of electron states are included in the numerical simulation, which nicely reproduces the experimental results. As shown in Fig. 2c and d, cold electrons, located at Γ valley with mobility of 380 cm^2^/V · s, contribute to the hot spot on the upper half of NW, while hot electrons, located at L valley with a mobility of 15 cm^2^/V · s (rigorously, equivalent mobility of below 30 cm^2^/V · s is estimated), lead to a hot spot on the lower half of NW. A more detailed discussion about the hot spot location and its dependence on electron mobility can be found in Supplementary Section 5.

**Figure 3.**
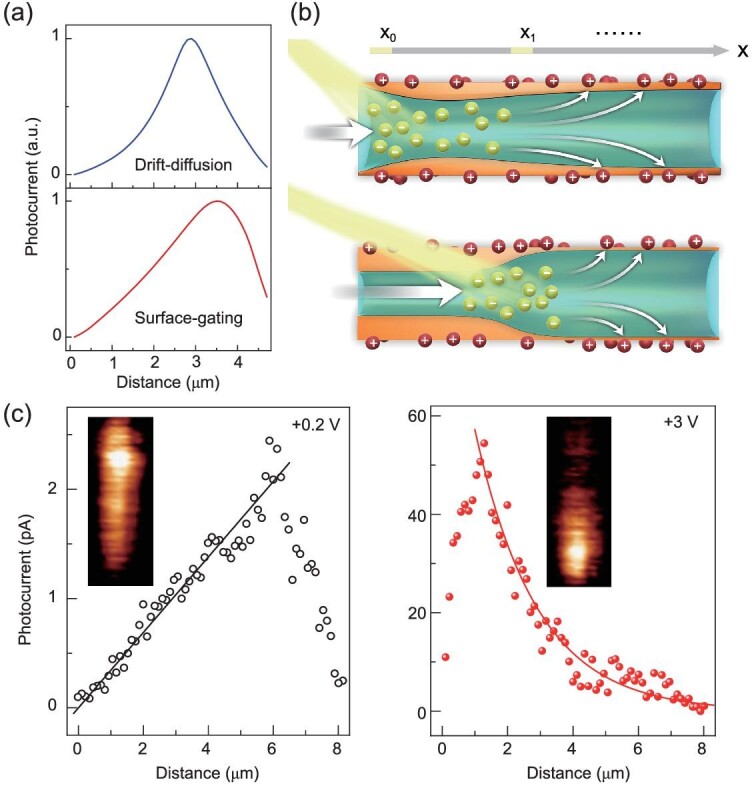
Carrier transport modes in GaAs NW transistor. (a) Simulated photocurrent profiles along NWs at *V*_ds _= 0.2 V by drift-diffusion model and surface-gating model, respectively. (b) Schematically showing the surface-effect-dominated carrier transport process when a laser spot is focused onto a small section of the NW. (c) Long channel (∼7.5 μm) GaAs NW transistor: photocurrent versus distance for NW biased at *V*_ds _= 0.2 V and *V*_ds_ = 3 V, respectively. Solid black (red) line is linearly (exponentially) fitted to the photocurrent profiles. The insets are corresponding SPCM images.

At this moment, the unique spatial resolved photoresponse properties of NW transistors have been resolved except for one thing: the origin of exponential and linear photocurrent decay profiles (Fig. 1f). To address this issue, we first focus on the classic drift-diffusion model. As stated earlier, there is only one solution for 1D photocarriers transport while electron and hole mobilities are fixed. In other words, photocarriers at a distance apart cannot contribute to the photocurrent of NW except by diffusing into the hot spot location, from where they will follow the 1D carrier transport rule (electrons and holes are simultaneously collected by anode and cathode, imposed by the charge neutrality principle). Since diffusion plays a dominant role in the process, an exponential dependence of photocarriers/photocurrent on spatial location is thus derived. The numerical simulation also supports this conclusion (Fig. 3a).

Then, the concept of photogating is introduced to interpret the linear decay profile at lower biases. As depicted in Fig. 3b, photocarriers are devoted to passivate the NW surface (an effect similar to a positive bias on the NW channel), instead of being collected by the terminal electrode. Herein, the laser-spot-center location is defined as *x_n_*. When a positive bias is applied to the right electrode, photocarriers (electrons) generated at *x_n_* position will be electrically swept to passivate the NW surface. But note that only those areas with *x *> *x_n_* will be photo-passivated, and the left-hand side (*x *< *x_n_*) is still in dark depletion status. In this case, the conductance of NW can be written as: $G\ = \frac{{{G_d}{x_n}}}{L}\ + \frac{{({G_d} + \Delta G)( {L - {x_n}} )}}{L}$, where ${G_d}$ is the conductance in dark condition, $L$ is the channel length, Δ$G$ is the peak photoconductance of the NW, $\frac{{{G_d}{x_n}}}{L}$ thus represents the conductance on the left-hand side of the laser spot, and $\frac{{({G_d} + \Delta G)( {L - {x_n}} )}}{L}$ stands for the conductance on the right-hand side of the laser spot. The equation can be simplified as $G\ = {G_d}\ + \Delta G - \frac{{\Delta G{x_n}}}{L}$, which then shows a linear dependence on the spatial location. Such characteristic is well-reproduced in numerical simulation (Fig. 3a, bottom panel), where the model is modified by adding fixed charges on the NW surface. According to the simulation, the photo-generated minority carriers are rapidly swept to passivate the NW surface, which subsequently suppresses the surface depletion and enhances the conductance. It is similar to a forward gate bias on the NW channel [34–37]. Such an effect directly leads to a spatially linear decay of photocurrent.

We further prepared a long channel (∼7.5 μm) transistor with SPCM images and profiles shown in Fig. 3c. Similarly, almost the entire channel has a photoresponse at *V*_ds _= 0.2 V while the photoresponse is highly localized at *V*_ds_ = 3 V, which is caused by slow linear decay (see the solid black fitting line in the left panel of Fig. 3c) and fast exponential decay (see the solid red fitting curve in the right panel of Fig. 3c), respectively. For an NW transistor, the driving force that tunes carrier transport from photogating to the traditional drift-diffusion model is the strong external electric field. Although the mobility has been significantly reduced at 5 V, the electron velocity is improved at least three times, and the density-of-states effective mass increases by 13.5 times, as compared with a 0.2 V biased case (Fig. 2b). It thus increases the probability of hot electrons escaping from surface trapping and finally traveling through the whole NW.

**Figure 4.**
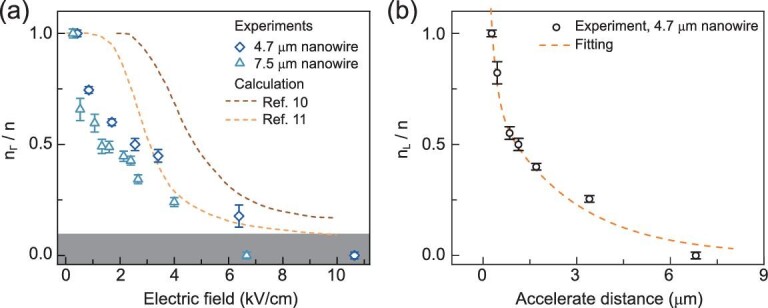
Quantifying electron population in the momentum-energy space. (a) Fractional ratio of electron population in the bottom Γ valley (Scatter lines, derived from the photocurrent ratio: ${I_A}/({I_A} + {I_B})$, where ${I_A}$ and ${I_B}$ are the peak photocurrent of feature A and B in Fig. 1f. Error bars: showing the deviation in extracting the photocurrent value. It is critical when both features coexist and a linear or exponential fit is used to distinguish their contributions.) and a comparison with the theoretical predictions (dashed lines). Orange dashed line: Reproduced with permission [10]. Copyright 1980, Elsevier, Ltd. Brown dashed line: Reproduced with permission [11]. Copyright 1968, American Physical Society. Note that calculations predict at least 10% of electrons left in the Γ valley at an electric field up to 10 kV/cm. This preserved region (marked in gray), however, is non-existent in experiments. (b) Ratio of electron population in L valley and its dependence on the accelerated distance (a distance that electrons have to accelerate before getting energy of 0.29 eV for climbing over the potential barrier between Γ and L valleys minimum). The dashed line is the fitting curve based on a double-exponential function: ${\rm{A\,exp}}( { - x/{L_1}} ) + {\rm{\ B\,exp}}( { - x/{L_2}} )$.

The distinct photoresponse observed here offers a unique opportunity to quantitatively resolve the carrier population and dynamic processes. As electrons located at Γ and L valleys are spatially separated from each other during the photoresponse process (A and B sites in Fig. 1f), the spatially-resolved photocurrent profile thus can be used to evaluate the occupation status of electrons in each valley. Specifically, ${I_A}/({I_A} + {I_B})$ is used to calculate the fractional ratio of the electron population in the bottom Γ valley, where ${I_A}$ and ${I_B}$ are the peak photocurrent of features A and B. For accuracy, linear and exponential fits are utilized to distinguish the photoresponse coming from Γ and L valley electrons, especially when both contributions coexist. Figure 4a shows the ratio of electron population in the Γ valley for 4.7 and 7.5 μm GaAs NWs. Obviously, those experimental results deviate from the theoretical predictions (from refs [10] and [11]), which are based on Monte Carlo methods for a weighted estimate of electron mobility and velocity. One distinguishing feature is that the Γ valley easily loses electrons, for example, ∼25% at an electric field of ∼0.5 kV/cm, which is 5–7 times earlier than the previous cognition. In other words, it is demonstrated that electrons are more easily warmed up and transferred to upper satellite valleys. We would like to emphasize another distinguishing feature in the experiments: Γ valley electrons are fully exhausted at an electric field of below 10 kV/cm. It deviates from the classical interpretation of carrier dynamics, where charge carrier scattering (by crystal lattice and defect atoms) is inevitable, leading to a balance between the upward transfer and downward relaxation processes. For this reason, a small percentage of electrons (10%–17%) are left in the bottom Γ valley. However, this preserved region (marked in gray in Fig. 4a) is non-existent in experiments. We attribute those behaviors to a much lower probability of scattering events for hot electrons. As theoretically predicted, the mean free path of electrons in the Γ valley is ∼150 nm, whereas the length scale is prolonged to ∼670 and ∼900 nm for L and X valley electrons [18]. It indicates that electrons at upper valleys are less sensitive to the scattering, explaining why they turn up earlier and very few of them relax to the bottom valley.

Figure 4b shows the electron population ratio for the L valley versus the accelerate distance (a distance that electrons have to accelerate before getting energy, 0.29 eV, to climb over the potential barrier between Γ and L valleys minimum). By fitting the curve with a double-exponential function: ${\rm{A\,exp}}( { - x/{L_1}} ) + {\rm{\ B\,exp}}( { - x/{L_2}} )$, short decay length scales (${L_1}$) of 180 and 170 nm are derived in 4.7 and 7.5 μm NW, respectively (the result of the 7.5 μm device is plotted in Supplementary Fig. S11). It is very close to the value of the electron mean free path of the bottom valley at room temperature (∼150 nm) [10]. Such character implies that electron acceleration within the range of a mean free path could be decisive for the carrier population and transport properties of GaAs NW.

**Figure 5.**
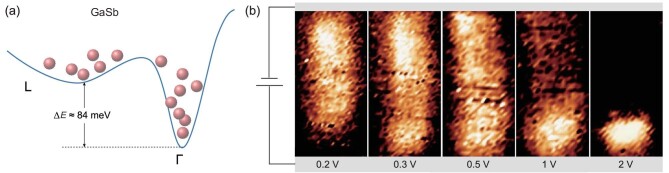
P-doped GaSb NW transistor. (a) Conduction band structure of GaSb with typical energy gap of ∼84 meV between Γ and L valleys. (b) SPCM images of GaSb NW (∼40 nm in diameter and ∼6 μm in channel length) at 0.2, 0.3, 0.5, 1 and 2 V, respectively.

III-V semiconductors possess a similar conduction band structure, where minimum Γ valley is surrounded by upper satellite ones, including X and L valleys [38]. Meanwhile, the nature of high mobility means charge carriers are easily warmed up, and the surface charge density is orders of magnitude higher than silicon and some other semiconductors [39]. These features indicate that carrier transport in III-V NW transistors could exhibit similar behaviors. To verify this, SPCM experiments on gallium antimonide NW (GaSb NW) were performed. GaSb NW characterized here is self-organized by chemical vapor deposition. The hole mobility is demonstrated up to 1028 cm^2^/V · s in our previous study [40]. As shown in Fig. 5b, the whole channel can respond to illumination at low bias while only sections close to the anode are photo-sensitive at large voltage, consistent with GaAs NW transistors (Fig. 3c). But note that the electric field required for the valley transfer is 2–3 times less (more results in supplementary information). It should arise from a ∼3 times decrease in the energy gap between Γ and L valleys as compared with GaAs. It is ∼84 and ∼290 meV for GaSb and GaAs, respectively [1,41]. It is worth noting that similar results are expected in a variety of material systems (not just limited to III-V semiconductor), once an intervalley scattering has been observed, including Ge, CdTe, InP, InAs, InSb and so on [42].

In summary, we comprehensively analyze the carrier dynamic and kinetic behavior induced by localized light excitation in a 1D NW structure under both the radial and axial electric fields, from both theoretical and experimental perspectives. In III-V semiconductor NW transistors, two competing hot spots are observed in SPCM images, which originate from cold and hot electrons, respectively, as a result of electron valley transfer and 1D carrier transport rule. This behavior clearly demonstrates the striking fact that hot electrons can be artificially (spatially) separated from cold ones in NW transistors. It allows us to verify when electrons are warmed up, where they locate, and how the electron occupied states, mobility, energy and the transport path evolve. Equally important, there is a direct observation on the microscopic process of the photogating effect in this study. For such effect, photocarriers contribute to the photoresponse by passivating the NW surface, which causes deviation from the drift-diffusion model. As a result, the photocurrent shows a linear dependence on the spatial location (deviating from the conventional exponential decay profile).

Finally, experiments indicate that hot electrons (in upper satellite valleys) possess a much longer mean free path compared with cold ones (in bottom Γ valleys). We highlight it for both electronic and optoelectronic applications. In a transistor, for example, electrons can be accelerated and scattered into upper valleys, where the long mean free path enables hot electrons to transit through the conduction channel with a low loss. Note that similar effects, like overshooting of hot electrons and non-local heat dissipation, have been observed in our previous study [43]. Together, those features might provide a particular perspective in reducing the power consumption and heat dissipation pressure of chips. Meanwhile, long-range hot-carrier transport is considered to have great potential to exceed the Shockley-Queisser limit of solar cells [44]. The concept of the hot-carrier solar cell has been proposed, where the cooling of photo-excited hot-carriers is intentionally slowed down, thus offering an opportunity to recover the energy loss (∼42%) due to thermalization [4]. It is conceptually superior to traditional architecture. However, there is still no clear route to realize such functionality. Very recently, Hamidreza *et al.* have reported the intervalley-scattering type solar cells, where photo-excited hot electrons are designed to occupy the upper valley states instead of relaxing to the bottom Γ valley [5]. Our study suggests that such a strategy may be much more feasible since hot electrons at upper satellite valleys are long-lived and show a much longer mean free path.

## METHODS

### GaAs NW synthesis

The GaAs NWs were grown on silicon (111) substrates using the Ga droplet self-catalysis growth method through an molecular beam epitaxy (MBE) system. Before the growth, the silicon wafer was treated for 30 s to remove the native oxide layer by a dilute hydrofluoric acid (HF) solution (12%) and loaded into the growth chamber immediately. The NW growth started with Ga deposition at 200°C for three monolayers (ML) to form Ga droplets. Then, the substrate was heated to 600°C for GaAs NW growth with a Ga growth rate of 0.3 ML/s under an As_4_ beam equivalent pressure (BEP) of ∼10^−6^ mbar. After the NW growth, Ga and As shutters were both closed completely and the substrate was cooled down to room temperature.

### GaSb NW synthesis

The GaSb NWs used in this work were synthesized on Si/SiO_2_ substrate through the surfactant-assisted solid-source CVD method. Before the NW growth, a 400 nm Sn film was deposited on the substrate, which took the responsibility of both catalyst and p-type dopants. In a dual-zone horizontal tube furnace, the GaSb powder source, substrate and sulfur surfactant were placed in the upstream zone, the downstream zone and the middle of two zones, respectively. NW growth started when the source temperature increased to 750°C and the substrate was heated to a temperature of 550°C. The pressure of the chamber in the growth process was kept at ∼3 × 10^−3^ Torr. High-purity hydrogen was employed as the carrier gas at a rate of 200 sccm.

After the growth, the heating of the source and substrate was turned off together and cooled down under the hydrogen flow.

### Device fabrication and characterization

Electron-beam lithography (EBL, JEOL 6510 with an NPGS System) was used to define the source/drain electrode. Before metallization, NWs were immersed into a 2% HF solution for approximately 5 s to remove the native oxide layer on the NW surface at room temperature and loaded into the chamber of thermal evaporation system immediately, ensuring the high-quality contact between metal electrodes and NWs. Next, NW transistors were fabricated by depositing Cr/Au metals and conducting lift-off processes. A semiconductor analyzer (Agilent-B1500) combined with a probe station (Lake Shore TTPX) was employed to perform *I*_ds_ − *V*_ds_ curve measurements in the dark and under the illumination of 450 nm.

### Device simulation

All numerical simulations for GaAs NWs in this work were performed using Sentaurus TCAD, a commercial package, where SDE and SDEVICE modules were employed to build up the 1D structure and carry out finite element calculation, respectively. With regard to the experimental setup, the diameter and length of p-type GaAs NW in the simulation were set to 100 nm and 4.7 μm; the values of electron/hole mobility and lifetime were set to 380/90 cm^2^/V · s and 0.5/1 ns (from refs [26] and [27]); the excitation light was set to 450 nm in wavelength and 1 μm in spot diameter. A p-type doping of 1 × 10^14^/cm^3^ for GaAs NW was considered. The spatial location of light spot was tunable to simulate the scanning behavior of laser-probe on NW. When the drift-diffusion model was considered, a coupled solution of Poisson, electron and hole continuity equations was performed. By contrast, the photogating effect was involved by additionally setting positive fixed charges of 4.5 × 10^10^/cm^2^ on the NW surface (note that the doping concentration was subsequently raised to 1 × 10^16^/cm^3^, which made sure that the static hole concentration was close to 1 × 10^14^/cm^3^, consistent with the counterpart of the drift-diffusion model).

## Supplementary Material

nwaa295_Supplemental_File
