# Supplementary material for: Direct observation and manipulation of hot electrons at room temperature
Source: Natl Sci Rev. 2020 Dec 15;8(9):nwaa295. doi: 10.1093/nsr/nwaa295 (PMC8433094; doi:10.1093/nsr/nwaa295)
Supplement: nwaa295_Supplemental_File [file nwaa295_supplemental_file.docx]

Supplementary Materials for

Direct observation and manipulation of hot electrons at room temperature

*Hailu Wang^1,5^*^†^*, Fang Wang^1,5^*^†^*, Hui Xia^1,5*^, Peng Wang^1,5,6^, Tianxin Li^1,5^, Juzhu Li^1^, Zhen Wang^1,5,6^, Jiamin Sun^2^, Peisong Wu^1,5^, Jiafu Ye^1,5^, Qiandong Zhuang^3^, Zaixing Yang^2^, Lan Fu^4^, Weida Hu^1,5^*, Xiaoshuang Chen^1,5*^, Wei Lu^1,5*^*

^1^State Key Laboratory of Infrared Physics, Shanghai Institute of Technical Physics, Chinese Academy of Sciences, 500 Yutian Road, Shanghai 200083, China

^2^School of Microelectronics, Shandong University, Jinan 250100, China

^3^Department of Physics, Lancaster University, Lancaster LA1 4YB, UK

^4^Department of Electronic Materials Engineering, Research School of Physics and Engineering, The Australian National University, Canberra, ACT 2601, Australia

^5^University of Chinese Academy of Sciences, Beijing 100049, China

^6^Henan Key Laboratory of Diamond Optoelectronic Materials and Devices, School of Physics and Engineering, Zhengzhou University, Zhengzhou 450001, China

*Corresponding author: [huix@mail.sitp.ac.cn](mailto:huix@mail.sitp.ac.cn); (H. Xia); [wdhu@mail.sitp.ac.cn](mailto:wdhu@mail.sitp.ac.cn) (W. Hu); [xschen@mail.sitp.ac.cn](mailto:xschen@mail.sitp.ac.cn); (X. Chen); [luwei@mail.sitp.ac.cn](mailto:luwei@mail.sitp.ac.cn). (W. Lu)

†H. Wang and F. Wang contributed equally to this work.

1. *SPCM images of the GaAs nanowire transistor with 4.7 μm channel length*


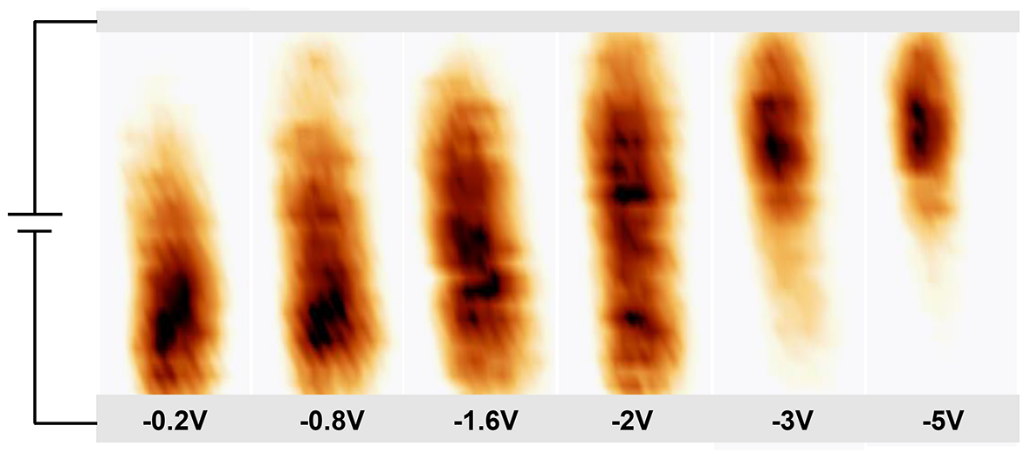


**Figure S1.** SPCM images of the GaAs nanowire transistor under negative bias-voltages on the bottom electrodes.

Non-uniform doping or asymmetric electrodes can strongly influence carrier dynamic and kinetic processes of semiconductor nanowire devices by generating local carrier modulation^1^. To exclude these effects, we further performed SPCM measurements of the GaAs nanowire transistor under negative bias-voltages, as shown in **Figure S1**. Consistent with the case under positive bias-voltages, a typical "hot spot" can be found to shift from near the cathode (through the entire nanowire channel) to the anode with an increasing negative bias-voltages. However, it should be noted that the turning point of ~4.3 kV/cm (-2 V) at which the electron drift velocity/mobility degrades is slightly different from that of positive bias-voltages (the turning point is 3.5 kV/cm at 1.6 V). This change can be attributed to the existence of a low contact barrier between electrodes and nanowire, but it doesn’t influence the conclusion of our work.

1. *SPCM images of the GaAs nanowire transistor with 7.5 μm channel length*


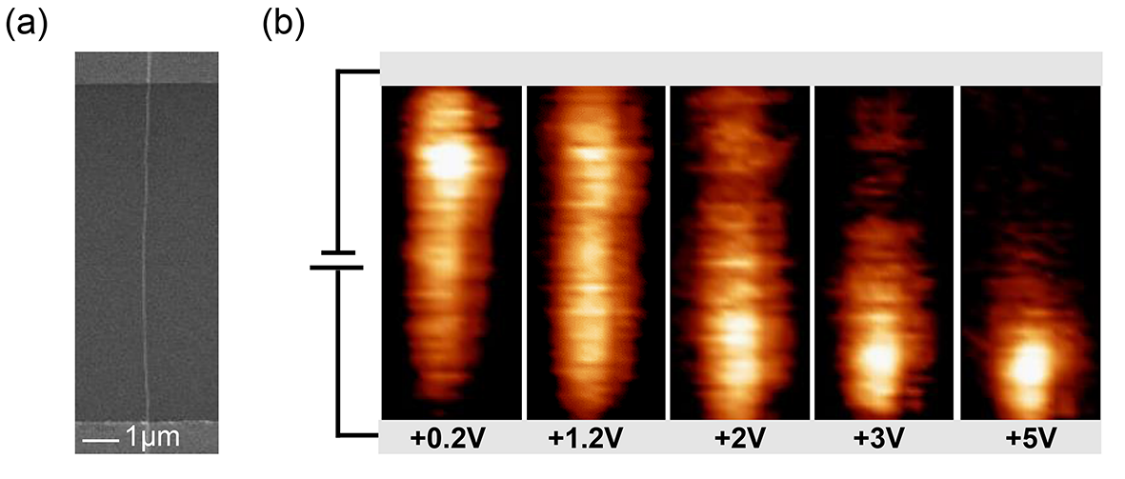


**Figure S2.** Long channel GaAs nanowire transistor. (a) SEM image of the GaAs nanowire transistor, with ~7.5 μm in channel length. (b) SPCM images at increasing bias-voltages.

**Figure S2b** displays SPCM images of a long channel (~7.5 μm) transistor. Typical “hot spots” in the SPCM patterns can be identified and shift from near the cathode to the anode with the increasing positive voltages. Furthermore, the entire channel has a photoresponse at low biases while the photoresponse is highly localized at high biases, consistent with the short channel transistor with ~4.7 μm in channel length.


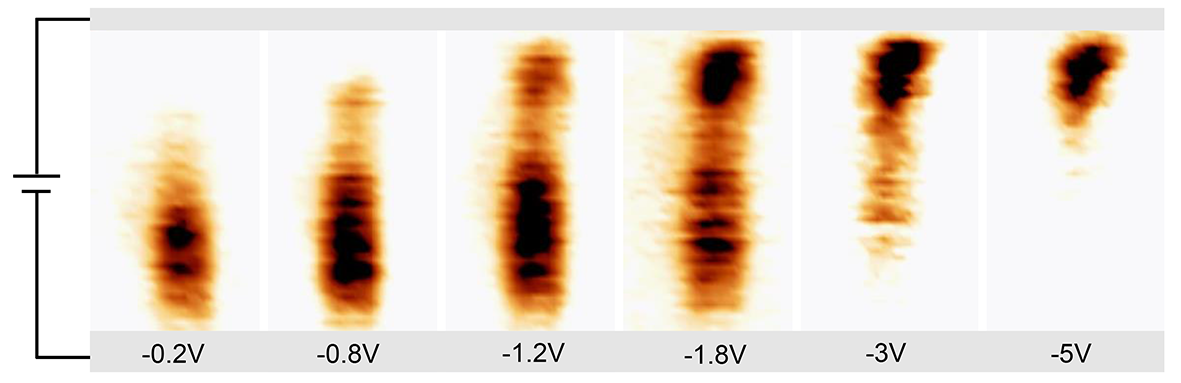


**Figure S3.** SPCM images of the GaAs nanowire transistor with ~7.5 μm channel length under negative bias-voltages on the bottom electrodes.

The negative bias-voltages have also been applied and corresponding SPCM patterns are shown in **Figure S3**.

**
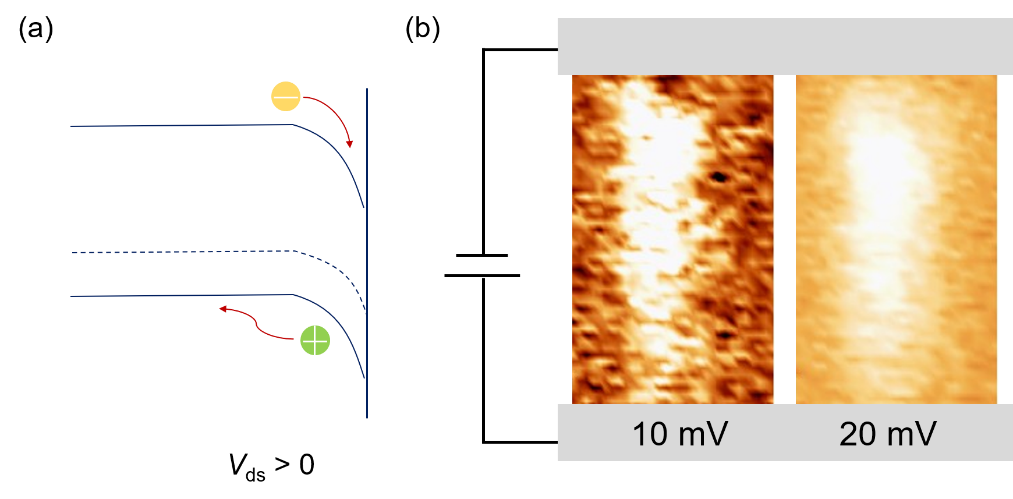
**

**Figure S4.** SPCM images under very small voltages. (a) Band structure at positive voltage. (b) SPCM images at 10 and 20 mV.

To exclude the influence of space charge region and contact-related photocurrent effects, we have also performed SPCM measurements under very small voltages, as shown in **Figure S4**.

1. *SPCM images of a long channel GaSb nanowire transistor*


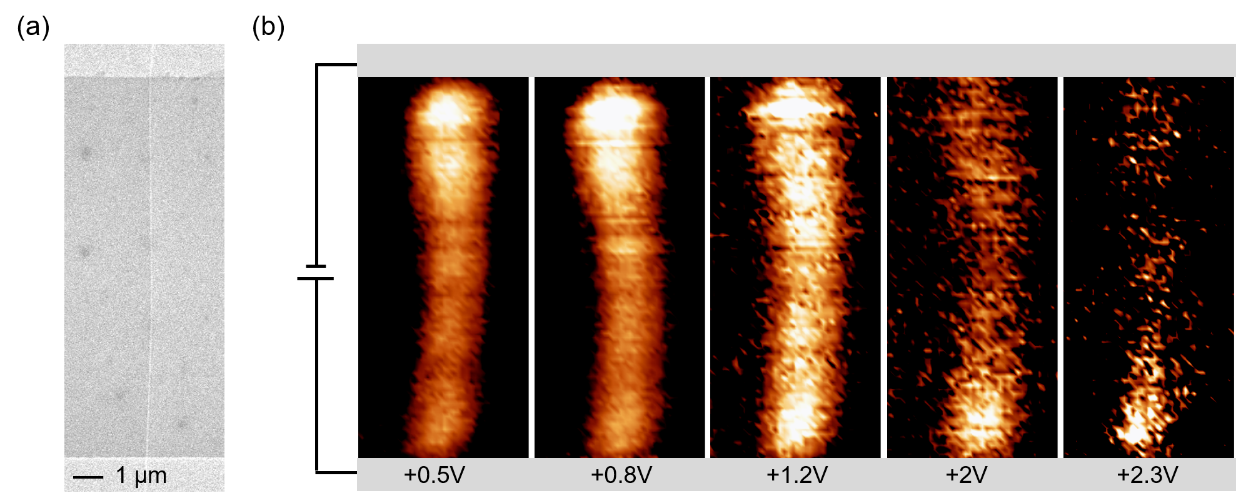


Figure S5. Long channel GaSb nanowire transistor. (a) SEM image of the GaSb nanowire transistor (~12 μm in channel length). (b) SPCM images of nanowire under increasing bias-voltages.

To verify that the conclusion in this work can be applied to other III-V semiconductors with similar band structure, we further prepared a longer channel (~12 μm) GaSb nanowire transistor and performed SPCM measurements. From **Figure S5b**, one can find that characteristics are consistent with GaAs nanowires. However, due to the smaller energy gap between Γ and L valleys (~84 meV for GaSb), the turning point of ~1 kV/cm (~1.2 V) is much smaller than that of GaAs.

1. *Electrical and photoelectric properties of a GaSb nanowire transistor*

*
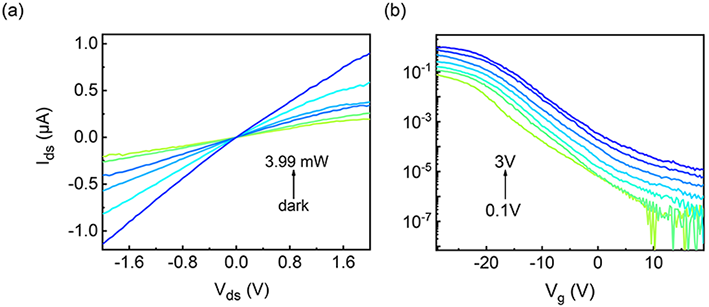
*

**Figure S6.** Electrical and photoelectric properties of a GaSb nanowire transistor. (a) Linear *I*_ds_-*V*_ds_ curves in dark and under a laser illumination of 450 nm. (b) Semi-logarithm *I*_ds_-*V*_g_ curves at increasing *V*_ds_ in dark.

We prepared GaSb nanowire transistors and performed electrical and photoelectric measurements, as indicated in **Figure S6a**. The relative linear *I*_ds_-*V*_ds_ curves in dark and under the illumination of 450 nm have demonstrated the good contact between GaSb nanowire and metal electrodes. Meanwhile, the transfer characteristic curves at increasing *V*_ds_ (**Figure S6b**) indicates the dominant role of holes in nanowire conductance. All measurements were performed at room temperature and atmospheric pressure.

1. *Dependence of hot spot location on the minority carrier (electron) mobility*

**Table S1.** Physical parameters employed in the **TCAD simulation.**

| NW material | doping characteristics | NW length | NW diameter | Electron mobility |
| --- | --- | --- | --- | --- |
| GaAs | 1×10^14^ cm^-3^ p-doped | 4.7 µm | 100 nm | 380 cm^2^/V·s ^a^ |
| electron lifetime | hole mobility | hole lifetime | laser wavelength | laser spot size |
| 0.5 ns ^a^ | 90 cm^2^/V·s ^a^ | 1 ns ^a^ | 450 nm | 1 µm |

Those values keep unchanged except for additional statement.

^a^ These values are taken from ref. 2 and 3 for p-doped GaAs nanowire.

Some key parameters of GaAs employed in the TCAD simulation are summarized in the **Table S1**. These values keep unchanged except for additional statement.


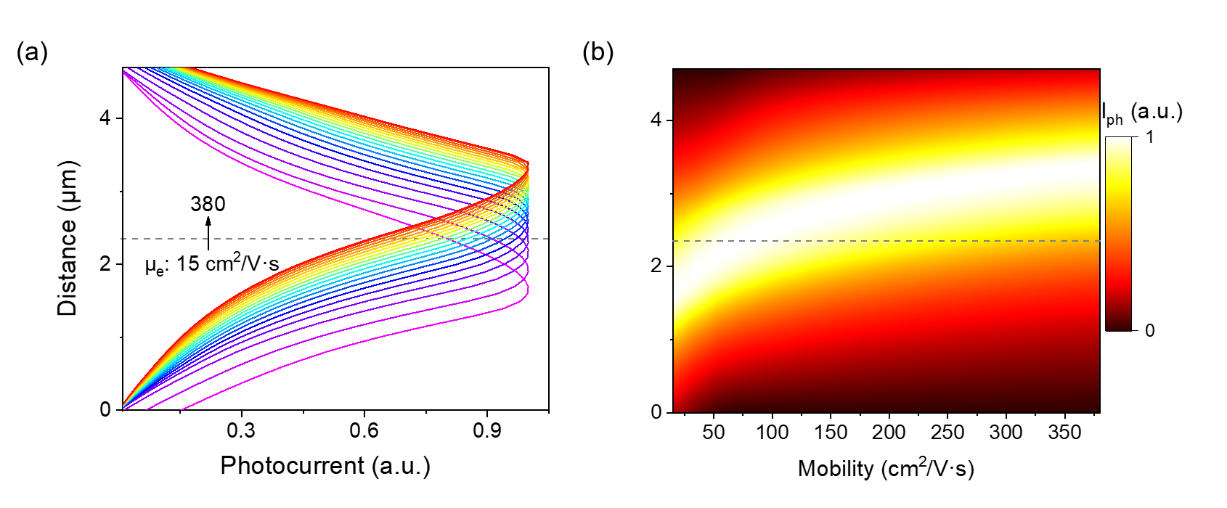


**Figure S7.** Dependence of photocurrent on electron mobility for the GaAs nanowire transistor with ~7.5 μm channel length. (a) Normalized photocurrent profiles along the nanowire with increasing electron mobility. (b) Photocurrent mapping images at *V*_ds_=1.6 V. The gray dashed lines represent the midpoint of the nanowire.

1. *Numerical simulation*

A numerical simulation was carried out to investigate how electron mobility influences the hot spot location employing the commercial package Sentaurus TCAD. In the simulation, the hole mobility was set to a constant (90 cm^2^/V·s) while electron mobility was set to vary from 15 cm^2^/V·s to 380 cm^2^/V·s considering the effect of electric field on electron mobility (other physical parameters keep unchanged, as listed in **Table S1**). From **Figure S7a**, one can find that photocurrent peaks shift gradually from the lower half of the nanowire to the upper half with increasing electron mobility. A “hot path” across the midpoint of the nanowire in the photocurrent mapping image (**Figure S7b**) intuitively indicates the valley-transfer, consistent with experimental results.


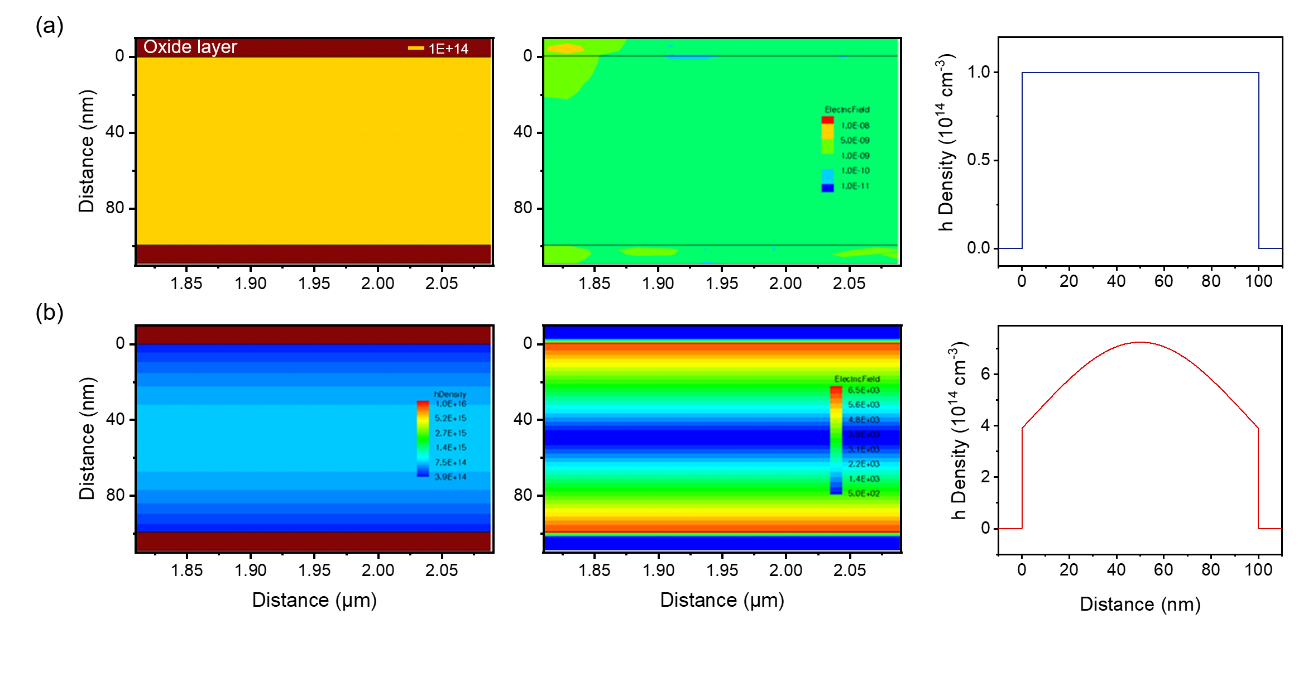


**Figure S8.** Device simulation of the GaAs nanowire transistor. The upper and lower panels are the case of setting (a) and not setting (b) positive fixed charges on the nanowire surface, respectively. First column: two-dimensional distribution of hole density. Second column: two-dimensional distribution of electric field intensity. Third column: hole density along the radial direction of the nanowire.

In the simulation, the diameter of nanowire and the thickness of the oxide layer on the nanowire surface was set to be 100 nm and 10 nm respectively. The p-type doping concentration of GaAs nanowire is 1×10^14^ /cm^3^. It should be noted that the doping concentration was subsequently raised to 1×10^16^ /cm^3^ considering the positive fixed charges of 4.5×10^10^ /cm^2^ on the nanowire surface, which ensures that the static hole concentration is close to 1×10^14^ /cm^3^.

1. *Quantifying electron population in the momentum-energy space*

*
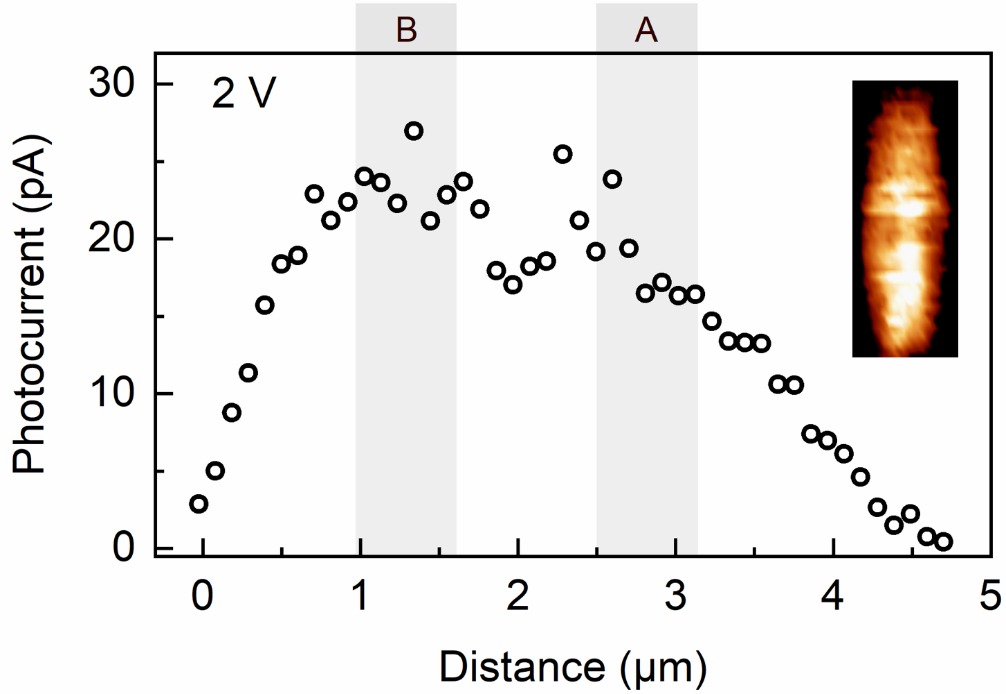
*

**Figure S9.** Photocurrent profile of the GaAs nanowire transistor with ~4.7 μm in channel length. The inset is the corresponding SPCM image at 2 V.

**Figure S9** indicates the photocurrent profile at 2 V and the corresponding SPCM pattern. Two peaks of photocurrent near locations A and B can be recognized clearly.





**Figure S10.** Photocurrent profiles of the long channel (~7.5 µm) GaAs nanowire extracted from corresponding SPCM images.

One-dimensional photocurrent profiles were taken from SPCM images at 0.2, 0.4, 0.8, 1, 1.2, 1.5, 1.8, 2, 3 and 5 V, respectively, shown in **Figure S10**. At small bias-voltage (0.2 V), there is only one peak of photocurrent which locates at near the cathode (denoted as feature A) while the other peak can be identified in the opposite direction (denoted as feature B) with the increasing of voltages. It should be noted that the peak of photocurrent near feature A disappears at 5 V, which indicates that electrons in the **Γ-**valley are fully transferred to the upper L-valley.





**Figure S11.** Ratio of electron population in the L-valley for the long channel (~7.5 µm) GaAs nanowire.

**Figure S11** shows the electron population ratio of the L-valley against the accelerate distance for the long channel (~7.5 µm) GaAs nanowire. To quantitatively study the carrier population, we calculated the ratio of electrons in the L-valley employing the equation ${I_{A}}/{{(I}_{A}+I_{B})}$, where $I_{A}$ and $I_{B}$ are the peak photocurrent of features A and B in **Figure S10**. The orange dashed line is the fitting curve using a double-exponential function: $\mathrm{Aexp} \left( -x/{L_{1}} \right)+ Bexp(-x/{L_{2}})$, a decay length scale ($L_{1}$) of 170 nm is derived.

1. *Experimental details of SPCM measurement setup*

*
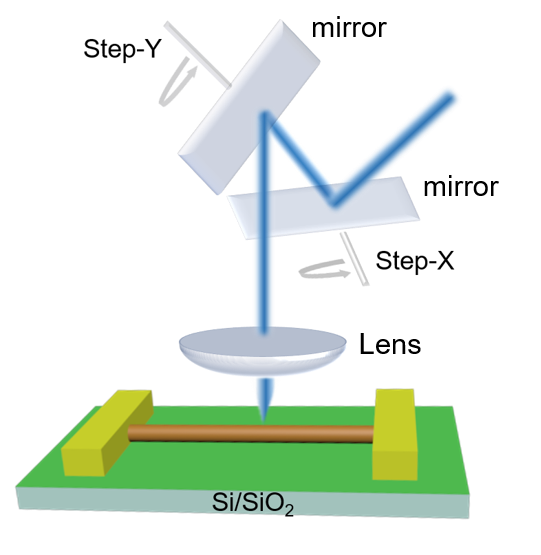
*

**Figure S12.** Schematic showing the SPCM measurement setup.

In the SPCM measurements, a fiber laser of 450 nm (Thorlabs LP450-SF15) modulated by a laser diode current and temperature controller (Thorlabs ITC4001) was focused on the devices using a microscope objective (Nikon ×100 0.9NA). During the measurements, the focused laser beam (~1 µm in diameter, 450 nm in wavelength, ~5 mW/cm^2^ in power density) was controlled by a scanning Galvo System (Thorlabs GVS212) to scan over the device area. The generated photocurrent signals were amplified by a Low-Noise Current Preamplifier (Stanford Research Systems SR570) in real-time, thereafter recorded using a lock-in amplifier (Signal Recovery model 7270).

Reference

1. Allen, J. E. *et al.* Nonuniform nanowire doping profiles revealed by quantitative scanning photocurrent microscopy. *Adv. Mater.* **21**, 3067-3072 (2009).
2. Boland, J. L. *et al.* Increased Photoconductivity Lifetime in GaAs Nanowires by Controlled n-Type and p-Type Doping. *ACS Nano* **10**, 4219-4227 (2016).
3. Gutsche, C. *et al.* Controllable p-type doping of GaAs nanowires during vapor-liquid-solid growth. *J. Appl. Phys.* **105**, 024305 (2009).
